# Supplementary material for: Vitamin D Metabolites in Nonmetastatic High-Risk Prostate Cancer Patients with and without Zoledronic Acid Treatment after Prostatectomy
Source: Cancers (Basel). 2022 Mar 18;14(6):1560. doi: 10.3390/cancers14061560 (PMC8946001; doi:10.3390/cancers14061560)
Supplement: Supplementary file 1 [file cancers-14-01560-s001.zip › cancers-1629933-supplementary.pdf]

cancers

MDPI

Supplementary Materials: Vitamin D Metabolites in Nonmetastatic High-Risk Prostate Cancer Patients with and without Zoledronic Acid Treatment after Prostatectomy

Carsten Stephan, Bernhard Ralla, Florian Bonn, Max Diesner, Michael Lein and Klaus Jung

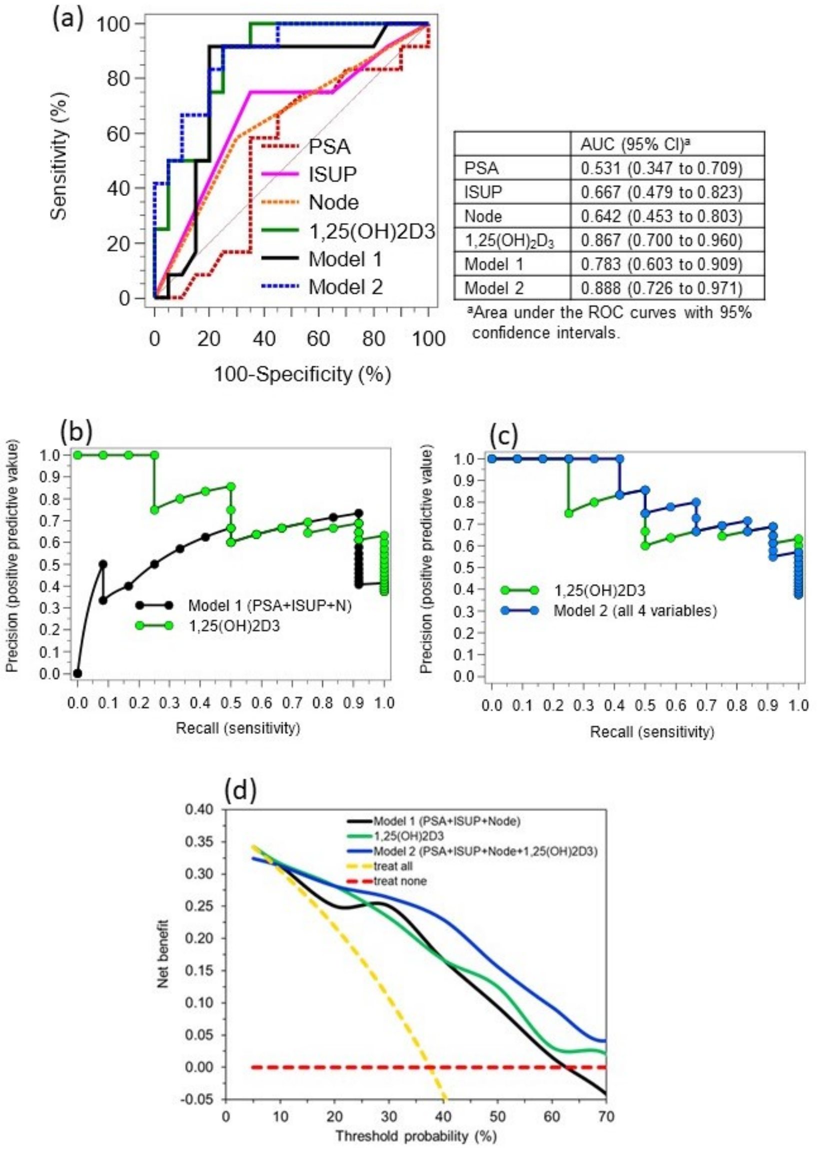

	AUC (95% CI)[a]
PSA	0.531 (0.347 to 0.709)
ISUP	0.667 (0.479 to 0.823)
Node	0.642 (0.453 to 0.803)
1,25(OH)$_2$D$_3$	0.867 (0.700 to 0.960)
Model 1	0.783 (0.603 to 0.909)
Model 2	0.888 (0.726 to 0.971)

[a]Area under the ROC curves with 95% confidence intervals.

Figure S1. 1,25(OH)$_2$D$_3$ level as prognostic indicator of subsequent bone metastasis after radical prostatectomy. (**a**) Receiver-operating characteristic curve analysis (ROC), (**b**,**c**) Precision-recall curve analysis, and (**d**) Decision-curve analysis. The prognostic validity of 1,25(OH)$_2$D$_3$ is indicated as single marker in comparison with the separate clinico-pathological factors prostate-specific antigen (PSA), Gleason scores as ISUP grade groups, and the Node (N) status as well as with the combination of these three variables (Model 1, calculated by binary logistic regression). Model 2 is based on all four variables (PSA, ISUP grade, Node status, and 1,25(OH)$_2$D$_3$).

Table S1. Clinicopathological risk factors of the study patients with and without zoledronic acid treatment and subsequent metastasis.

Pat_ID	PSA at diagnosis (µg/L)	Gleason score 1 + Gleason score 2	Node positive disease	Numer of risk factors	PSA before study entry (µg/L)	ZA treatment	Subsequent metastasis
D001	4	4+5	No	1	<0.03	No	No
D002	6	4+5	No	1	<0.03	No	No
D003	28	3+4	Yes	2	0.04	Yes	No
D004	22	4+3	No	1	1.26	No	Yes
D005	18	5+4	Yes	2	0.07	No	No
D006	15	4+4	No	1	0	No	No
D007	19	4+5	Yes	2	9.99	Yes	No
D008	7	4+5	No	1	0.47	Yes	Yes
D009	25	5+4	No	2	0.17	Yes	No
D010	13	4+5	No	1	0.11	Yes	Yes
D011	31	4+3	No	1	0.18	Yes	No
D012	7	3+5	No	1	0	No	No
D013	18	4+4	No	1	0.03	Yes	No
D014	18	4+5	Yes	2	0.89	No	Yes
D015	141	3+4	Yes	2	1.12	Yes	Yes
D016	49	4+5	Yes	3	0.88	No	No
D017	21	4+4	NA	2	0.03	No	No
D018	33	3+4	No	1	2.4	No	No
D019	13	5+4	No	1	0.29	No	Yes
D020	5	4+5	Yes	2	5.1	No	Yes
D021	51	4+3	No	1	<0.1	No	No
D022	15	3+4	Yes	1	0.8	No	No
D023	22	3+3	No	1	21.5	Yes	No
D024	17	5+4	No	1	1.5	No	Yes
D025	47	4+3	Yes	2	0.25	Yes	Yes
D026	13	4+5	Yes	2	0.41	Yes	Yes
D027	3	4+5	No	1	<0.04	Yes	No
D028	5	4+4	No	1	0.04	Yes	No
D029	9	3+4	Yes	1	<0.002	Yes	No
D030	9	4+4	No	1	ND	Yes	No
D031	14	4+5	Yes	2	0.019	Yes	Yes
D032	10	4+5	Yes	2	0.4	Yes	Yes

NA = not available, regional lymph nodes could not be assessed. ND = not determined.ZA treatment = Yes, patients treated with zoledronic acid; No, patients who did not receive zoledronic acid.
